# Supplementary material for: Seroepidemiology of SARS-CoV-2 in healthcare personnel working at the largest tertiary COVID-19 referral hospitals in Mexico City
Source: PLoS One. 2022 Mar 17;17(3):e0264964. doi: 10.1371/journal.pone.0264964 (PMC8929624; doi:10.1371/journal.pone.0264964)
Supplement: S1 Appendix — (DOCX) [file pone.0264964.s001.docx]

**S1 Appendix**

**C35-20 protocol questionnaire**

| Participant Information | |
| --- | --- |
| First name |  |
| Last name |  |
| Gender | □ Male □ Female □ Other / Unknown |
| Date of birth (DD / MM / YYYY) | ___/___/___ |
| Age (Years) |  |
| Telephone (10-digit) |  |
| E-mail |  |
| State |  |
| Municipality |  |
| Institution | INER  INCMNSZ |
| Occupation | □ Doctor  □ Nurse / Nursing Assistant  □ Radiologist / Imaging Technician  □ Inhalation therapist  □ Physiotherapist  □ Nutritionist  □ Stretcher-bearer  □ Cleanliness staff  □ Laboratory staff  □ Security staff  □ Researcher / Graduate student  □ Administrative staff |
| Recent contact (last 15 days) with any person diagnosed with COVID-19 | □ Yes  □ No  □ Unknown |
| Management of COVID-19 samples | □ Yes  □ No  □ Unknown |
| Contact with COVID-19 patients | □ No contact  □ Occasionally  □ Regularly |
| Use of PPE at work | □ Always  □ Generally  □ Sometimes  □ Never |
| Hand washing resources availability | □ Always  □ Generally  □ Sometimes  □ Never |
| Physical distancing availability | □ Always  □ Generally  □ Sometimes  □ Never |
| Use of public transport for commute to work | □ Yes  □ No  If the answer is “Yes”, what means of transportation (check all that apply)?□ Metro  □ Minibus / Truck / Combi  □ Taxi  □ Uber or another transportation app |

| Symptoms History | |
| --- | --- |
| Have you had any of the following symptoms throughout the COVID-19 pandemic? | |
| Fever (≥38°C) | □ Yes □ No |
| Chills | □ Yes □ No |
| Fatigue | □ Yes □ No |
| Muscular pain | □ Yes □ No |
| Sore throat | □ Yes □ No |
| Cough | □ Yes □ No |
| Rhinorrhea (runny nose) | □ Yes □ No |
| Dyspnea (hard breathing) | □ Yes □ No |
| Chest pain | □ Yes □ No |
| Other respiratory symptoms | □ Yes □ No |
| Headache | □ Yes □ No |
| Nausea | □ Yes □ No |
| Abdominal pain | □ Yes □ No |
| Diarrhea | □ Yes □ No |
| Anosmia (loss of smell) | □ Yes □ No |
| Ageusia (loss of taste) | □ Yes □ No |
| Medical care needed | □ Yes □ No □ Unknown |
| Work absenteeism because of symptoms | □ Yes □ No □ Unknown |
| Hospitalization | □ Yes □ No □ Unknown |
