# Supplementary material for: Seroepidemiology of SARS-CoV-2 in healthcare personnel working at the largest tertiary COVID-19 referral hospitals in Mexico City
Source: PLoS One. 2022 Mar 17;17(3):e0264964. doi: 10.1371/journal.pone.0264964 (PMC8929624; doi:10.1371/journal.pone.0264964)
Supplement: S1 Table — (DOCX) [file pone.0264964.s003.docx]

**S1 Table. Variation in the number of participants at baseline and follow-up along the study period.**

|  | Sample 1 | | Sample 2 | | Sample 3 | | Sample 4 | | Sample 5 | |
| --- | --- | --- | --- | --- | --- | --- | --- | --- | --- | --- |
| Total of participants, n (%) | 883 | (100) | 774 | (88) | 622 | (70) | 467 | (53) | 280 | (32) |
| Total positive samples, n (%) | 235 | (27) | 235 | (30) | 194 | (31) | 148 | (32) | 86 | (31) |
| New positive samples, n (%) | 235 | (27) | 30 | (4) | 15 | (2) | 7 | (2) | 3 | (1) |
|  |  | | Difference between samples 1 and 2 | | Difference between samples 2 and 3 | | Difference between samples 3 and 4 | | Difference between samples 4 and 5 | |
| Days between samples, median (IQR) |  |  | 29 | (23 – 42) | 29 | (23 – 40) | 30 | (24 – 42) | 32 | (26 – 44) |
| Lost-to-follow-up, n (%) |  |  | 109 | (12) | 152 | (20) | 15 | (25) | 187 | (40) |
