# Supplementary material for: Seroepidemiology of SARS-CoV-2 in healthcare personnel working at the largest tertiary COVID-19 referral hospitals in Mexico City
Source: PLoS One. 2022 Mar 17;17(3):e0264964. doi: 10.1371/journal.pone.0264964 (PMC8929624; doi:10.1371/journal.pone.0264964)
Supplement: S2 Table — (DOCX) [file pone.0264964.s004.docx]

**S2 Table. Symptoms reported by exposure group in healthcare workers of the two largest COVID-19 referral hospitals in Mexico City, October 2020-June 2021.**

| Symptom | Non-cases  (n=593) | | Prevalent cases  (n=235) | | Incident cases  (n=55) | |  | *P* value |  |
| --- | --- | --- | --- | --- | --- | --- | --- | --- | --- |
|  | n | (%) | n | (%) | n | (%) | ^a^ | ^b^ | ^c^ |
| Fever | 50 | (8) | 81 | (35) | 7 | (13) | **<0.001** | 0.28 | **0.002** |
| Chills | 90 | (15) | 107 | (46) | 11 | (20) | **<0.001** | 0.35 | **0.001** |
| Fatigue | 235 | (40) | 152 | (65) | 33 | (60) | **<0.001** | **0.003** | 0.52 |
| Myalgia | 180 | (30) | 147 | (63) | 24 | (44) | **<0.001** | **0.04** | **0.01** |
| Sore throat | 262 | (44) | 134 | (57) | 25 | (45) | **0.001** | 0.86 | 0.12 |
| Cough | 123 | (21) | 106 | (45) | 21 | (38) | **<0.001** | **0.003** | 0.35 |
| Rhinorrhea | 246 | (41) | 105 | (45) | 26 | (47) | 0.40 | 0.41 | 0.73 |
| Dyspnoea | 36 | (6) | 55 | (23) | 9 | (16) | **<0.001** | **0.004** | 0.26 |
| Thoracic pain | 48 | (8) | 66 | (28) | 12 | (21) | **<0.001** | **0.001** | 0.35 |
| Other respiratory symptoms | 42 | (7) | 35 | (15) | 2 | (4) | **<0.001** | 0.33 | **0.02** |
| Headache | 314 | (53) | 153 | (65) | 28 | (51) | **0.001** | 0.77 | **0.05** |
| Nausea | 72 | (12) | 46 | (20) | 11 | (20) | **0.006** | 0.10 | 0.94 |
| Abdominal pain | 76 | (13) | 48 | (20) | 6 | (11) | **0.006** | 0.68 | 0.10 |
| Diarrhea | 149 | (25) | 84 | (36) | 8 | (15) | **0.002** | 0.08 | **0.002** |
| Anosmia | 20 | (3) | 119 | (51) | 19 | (35) | **<0.001** | **<0.001** | **0.03** |
| Ageusia | 16 | (3) | 109 | (46) | 14 | (25) | **<0.001** | **<0.001** | **0.005** |
| Sought medical care | 107 | (18) | 127 | (54) |  |  | **<0.001** |  |  |
| Missed work days | 87 | (15) | 132 | (56) |  |  | **<0.001** |  |  |
| Required hospitalization | 5 | (1) | 15 | (6) |  |  | **<0.001** |  |  |

^a^ Prevalent cases vs. non-cases; ^b^ Incident cases vs. non-cases; ^c^ incident vs. prevalent cases.
