# Supplementary material for: Seroepidemiology of SARS-CoV-2 in healthcare personnel working at the largest tertiary COVID-19 referral hospitals in Mexico City
Source: PLoS One. 2022 Mar 17;17(3):e0264964. doi: 10.1371/journal.pone.0264964 (PMC8929624; doi:10.1371/journal.pone.0264964)
Supplement: S3 Table — (DOCX) [file pone.0264964.s005.docx]

**S3 Table. Risk factors by occupation in prevalent cases, October 2020-June 2021.^a^**

|  | | Physician | | Nurse | | Laboratory Technician | | Administrative | | Other | |  |
| --- | --- | --- | --- | --- | --- | --- | --- | --- | --- | --- | --- | --- |
|  |  | **n** | **(%)** | **n** | **(%)** | **n** | **(%)** | **n** | **(%)** | **n** | **(%)** | ***P* value^d^** |
| Contact with any person | No/Unknown | 8 | (17) | 13 | (17) | 6 | (38) | 24 | (55) | 18 | (36) | <0.001 |
| with COVID-19^b^ | Yes | 39 | (83) | 63 | (83) | 10 | (63) | 20 | (45) | 32 | (64) |  |
| Handling of biological | No/Unknown | 11 | (23) | 20 | (26) | 5 | (31) | 43 | (98) | 35 | (70) | <0.001 |
| specimens | Yes | 36 | (77) | 56 | (74) | 11 | (69) | 1 | (2) | 15 | (30) |  |
| Contact with patients | Never/Occasionally | 4 | (9) | 11 | (14) | 8 | (50) | 32 | (73) | 20 | (40) | <0.001 |
| with COVID-19^c^ | Frequently | 43 | (91) | 65 | (86) | 8 | (50) | 12 | (27) | 30 | (60) |  |
| Use of PPE | Never | 1 | (2) | 5 | (7) | 0 | (0) | 7 | (16) | 1 | (2) | 0.23 |
|  | Always/Generally | 46 | (98) | 71 | (93) | 16 | (100) | 37 | (84) | 49 | (98) |  |
| Use of face mask | Never/Sometimes | 0 | (0) | 1 | (1) | 0 | (0) | 0 | (0) | 0 | (0) | 0.72 |
|  | Always/Generally | 47 | (100) | 75 | (99) | 16 | (100) | 44 | (100) | 50 | (100) |  |
| Hand washing | No | 0 | (0) | 1 | (1) | 0 | (0) | 0 | (0) | 0 | (0) | 0.72 |
|  | Yes | 47 | (100) | 75 | (99) | 16 | (100) | 44 | (100) | 50 | (100) |  |
| PPE, personal protection equipment; ^a^ Total prevalent cases = 235, column percentages are shown; ^b^ Suspected or confirmed during the last 15 days; ^c^ Suspected or confirmed since March 2020; ^d^ Chi-square test, two-sided P values are shown ^c^ insufficient observations. | | | | | | | | | | | | |
