# Supplementary material for: Seroepidemiology of SARS-CoV-2 in healthcare personnel working at the largest tertiary COVID-19 referral hospitals in Mexico City
Source: PLoS One. 2022 Mar 17;17(3):e0264964. doi: 10.1371/journal.pone.0264964 (PMC8929624; doi:10.1371/journal.pone.0264964)
Supplement: S4 Table — (DOCX) [file pone.0264964.s006.docx]

**S4 Table. Risk factors by occupation in incident cases, October 2020-June 2021.**^a^

|  | | Physician | | Nurse | | Administrative | | Other | |  |
| --- | --- | --- | --- | --- | --- | --- | --- | --- | --- | --- |
|  |  | **n** | **(%)** | **n** | **(%)** | **n** | **(%)** | **n** | **(%)** | ***P* value^d^** |
| Contact with any person | No/Unknown | 6 | (40) | 2 | (13) | 11 | (58) | 2 | (50) | 0.05 |
| with COVID-19^b^ | Yes | 9 | (60) | 14 | (88) | 8 | (42) | 2 | (50) |  |
| Handling of biological | No/Unknown | 10 | (67) | 8 | (50) | 16 | (84) | 4 | (100) | 0.08 |
| specimens | Yes | 5 | (33) | 8 | (50) | 3 | (16) | 0 | (0) |  |
| Contact with patients with | Never/Occasionally | 6 | (40) | 7 | (44) | 17 | (89) | 2 | (50) | 0.01 |
| with COVID-19^c^ | Frequently | 9 | (60) | 9 | (56) | 2 | (11) | 2 | (50) |  |
| Use of PPE | Never | 1 | (7) | 1 | (6) | 4 | (21) | 0 | (0) | 0.10 |
|  | Always/Generally | 14 | (93) | 15 | (94) | 15 | (79) | 4 | (100) |  |
| Use of face mask | Never/Sometimes | 1 | (7) | 1 | (6) | 0 | (0) | 0 | (0) | 0.67 |
|  | Always/Generally | 14 | (93) | 15 | (94) | 19 | (100) | 4 | (100) |  |
| Hand washing | No | 0 | (0) | 0 | (0) | 0 | (0) | 0 | (0) | ^e^ |
|  | Yes | 15 | (100) | 16 | (100) | 19 | (100) | 4 | (100) |  |
| PPE, personal protection equipment; ^a^ Total incident cases = 55, column percentages are shown; ^b^ Suspected or confirmed during the last 15 days; ^c^ Suspected or confirmed since March 2020; ^d^ Chi-square test, two-sided P values are shown; ^e^ Insufficient observations. | | | | | | | | | | |
