# Supplementary material for: Seroepidemiology of SARS-CoV-2 in healthcare personnel working at the largest tertiary COVID-19 referral hospitals in Mexico City
Source: PLoS One. 2022 Mar 17;17(3):e0264964. doi: 10.1371/journal.pone.0264964 (PMC8929624; doi:10.1371/journal.pone.0264964)
Supplement: S5 Table — (DOCX) [file pone.0264964.s007.docx]

**S5 Table. Differences between persons who did and did not attend at least one visit for blood sample donation for antibody testing that registered in the study database of healthcare workers of the two largest COVID-19 referral hospitals in Mexico City, October 2020-June 2021.**

|  |  | Without antibody tests  (n=246) | | With at least one  antibody test  (n=883) | | *P* value^a^ |
| --- | --- | --- | --- | --- | --- | --- |
| Sex, n (%) | Male | 87 | (35.4) | 262 | (29.7) | 0.09 |
|  | Female | 159 | (64.6) | 620 | (70.3) |  |
| Age, median (IQR) |  | 33 | (27-43) | 36 | (28-46) | 0.03 |
| Institution, n (%) | INER | 135 | (54.9) | 548 | (62.1) | 0.04 |
|  | INCMNSZ | 111 | (45.1) | 335 | (37.9) |  |
| State of residency, n (%) | Mexico City | 201 | (83.4) | 752 | (85.9) | 0.39 |
|  | State of Mexico | 35 | (14.5) | 100 | (11.4) |  |
|  | Other | 5 | (2.1) | 23 | (2.6) |  |
| Municipality, n (%) | Tlalpan | 74 | (37.6) | 283 | (37.8) | 0.43 |
|  | Coyoacán | 20 | (10.2) | 108 | (14.4) |  |
|  | Xochimilco | 25 | (12.7) | 71 | (9.5) |  |
|  | Iztapalapa | 18 | (9.1) | 63 | (8.4) |  |
|  | Other^b^ | 60 | (30.5) | 223 | (29.8) |  |
| Occupation, n (%) | Medical Doctor | 52 | (21.1) | 212 | (24.2) | <0.001 |
|  | Nurse | 101 | (41.1) | 244 | (27.8) |  |
|  | Laboratory technician | 3 | (1.2) | 38 | (4.3) |  |
|  | Administrative | 38 | (15.5) | 195 | (22.2) |  |
|  | Other | 52 | (21.1) | 189 | (21.5) |  |
| Previous COVID-19 diagnosis, n (%) | No | 113 | (86.9) | 681 | (77.3) | 0.04 |
|  | Yes | 16 | (12.3) | 187 | (21.2) |  |
|  | Unknown | 1 | (0.8) | 13 | (1.5) |  |
| Contact with any person with COVID-19, n (%)^c^ | No | 37 | (28.5) | 338 | (38.4) | 0.08 |
|  | Yes | 84 | (64.6) | 480 | (54.5) |  |
|  | Unknown | 9 | (6.9) | 63 | (7.2) |  |
| Handling of biological specimens, n (%) | No | 62 | (47.7) | 549 | (62.3) | 0.002 |
|  | Yes | 65 | (50.0) | 326 | (37.0) |  |
|  | Unknown | 3 | (2.3) | 6 | (0.7) |  |
| Contact with patients with COVID-19, n (%) | Never | 27 | (20.8) | 325 | (36.9) | <0.001 |
|  | Occasionally | 27 | (20.8) | 189 | (21.5) |  |
|  | Frequently | 76 | (58.5) | 367 | (41.7) |  |
| Use of PPE, n (%) | Never | 5 | (3.9) | 36 | (4.1) | 0.70 |
|  | Always / Generally | 122 | (93.9) | 812 | (92.2) |  |
|  | Sometimes | 3 | (2.3) | 33 | (3.8) |  |
| Use of face mask, n (%) | Never | 1 | (0.8) | 6 | (0.7) | 0.89 |
|  | Always / Generally | 129 | (99.2) | 875 | (99.3) |  |
| Use of public transport, n (%) | No | 47 | (36.2) | 455 | (51.7) | 0.001 |
|  | Yes | 83 | (63.9) | 426 | (48.4) |  |
| INCMNSZ, National Institute of Medical Sciences and Nutrition; INER, National Institute of Respiratory Diseases; IQR, interquartile range; PPE, Personal Protection Equipment. ^a^ Chi-square test, two-sided *P* values are shown; ^b^ Includes other municipalities in Mexico City, as well as municipalities in other states; ^c^ Suspected or confirmed cases from March 2020. | | | | | | |
