# Supplementary material for: Seroepidemiology of SARS-CoV-2 in healthcare personnel working at the largest tertiary COVID-19 referral hospitals in Mexico City
Source: PLoS One. 2022 Mar 17;17(3):e0264964. doi: 10.1371/journal.pone.0264964 (PMC8929624; doi:10.1371/journal.pone.0264964)
Supplement: Attachment — Submitted filename: Response to Reviewers.docx [file pone.0264964.s008.docx]

**Journal Requirements:**

When submitting your revision, we need you to address these additional requirements.

1. Please ensure that your manuscript meets PLOS ONE's style requirements, including those for file naming. The PLOS ONE style templates can be found at

<https://journals.plos.org/plosone/s/file?id=wjVg/PLOSOne_formatting_sample_main_body.pdf> and <https://journals.plos.org/plosone/s/file?id=ba62/PLOSOne_formatting_sample_title_authors_affiliations.pdf>.

**A: The manuscript has been formatted to meet PLoS One’s style requirements.**

2. Please include additional information regarding the survey or questionnaire used in the study and ensure that you have provided sufficient details that others could replicate the analyses. For instance, if you developed a questionnaire as part of this study and it is not under a copyright more restrictive than CC-BY, please include a copy, in both the original language and English, as Supporting Information.

**A: The questionnaire has been added as a supplementary file.**

3. Thank you for stating the following in the Acknowledgments Section of your manuscript:

“This work was supported by Consejo Nacional de Ciencia y Tecnología (CONACyT) (Fondo FORDECYT-PRONACES) and the Mexican Government (Programa Presupuestal P016; Anexo 13 del Decreto del Presupuesto de Egresos de la Federación) to SAR. Funders had no role in study design, data collection, analysis or interpretation, writing of the report and decision to submit for publication.”

We note that you have provided additional information within the Acknowledgements Section that is not currently declared in your Funding Statement. Please note that funding information should not appear in the Acknowledgments section or other areas of your manuscript. We will only publish funding information present in the Funding Statement section of the online submission form.

Please remove any funding-related text from the manuscript and let us know how you would like to update your Funding Statement. Currently, your Funding Statement reads as follows:

“This work was supported by Consejo Nacional de Ciencia y Tecnología (CONACyT) (Fondo FORDECYT-PRONACES) and the Mexican Government (Programa Presupuestal P016; Anexo 13 del Decreto del Presupuesto de Egresos de la Federación) to SAR. Funders had no role in study design, data collection, analysis or interpretation, writing of the report and decision to submit for publication.”

Please include your amended statements within your cover letter; we will change the online submission form on your behalf.

**A: We confirm that the information included in the Funding Statement is correct. We have removed this information from the Acknowledgements section.**

4. We note that you have indicated that data from this study are available upon request. PLOS only allows data to be available upon request if there are legal or ethical restrictions on sharing data publicly. For more information on unacceptable data access restrictions, please see <http://journals.plos.org/plosone/s/data-availability#loc-unacceptable-data-access-restrictions>.

In your revised cover letter, please address the following prompts:

a) If there are ethical or legal restrictions on sharing a de-identified data set, please explain them in detail (e.g., data contain potentially sensitive information, data are owned by a third-party organization, etc.) and who has imposed them (e.g., an ethics committee). Please also provide contact information for a data access committee, ethics committee, or other institutional body to which data requests may be sent.

b) If there are no restrictions, please upload the minimal anonymized data set necessary to replicate your study findings as either Supporting Information files or to a stable, public repository and provide us with the relevant URLs, DOIs, or accession numbers. For a list of acceptable repositories, please see<http://journals.plos.org/plosone/s/data-availability#loc-recommended-repositories>.

We will update your Data Availability statement on your behalf to reflect the information you provide

**A: We have included the anonymized dataset used for the analyses as supporting information.**

5. Please include your full ethics statement in the ‘Methods’ section of your manuscript file. In your statement, please include the full name of the IRB or ethics committee who approved or waived your study, as well as whether or not you obtained informed written or verbal consent. If consent was waived for your study, please include this information in your statement as well.

**A: The full ethics statement is now included in the Methods, including data on the IRB who approved the study.**

6. Your ethics statement should only appear in the Methods section of your manuscript. If your ethics statement is written in any section besides the Methods, please move it to the Methods section and delete it from any other section. Please ensure that your ethics statement is included in your manuscript, as the ethics statement entered into the online submission form will not be published alongside your manuscript.

**A: The ethics statement has been moved to the Methods and removed from other sections of the manuscript.**

**Reviewers' comments:**

**Reviewer's Responses to Questions**

1. Is the manuscript technically sound, and do the data support the conclusions?

The manuscript must describe a technically sound piece of scientific research with data that supports the conclusions. Experiments must have been conducted rigorously, with appropriate controls, replication, and sample sizes. The conclusions must be drawn appropriately based on the data presented.

Reviewer #1: Yes

Reviewer #2: Yes

2. Has the statistical analysis been performed appropriately and rigorously?

Reviewer #1: Yes

Reviewer #2: Yes

3. Have the authors made all data underlying the findings in their manuscript fully available?

The [PLOS Data policy](http://www.plosone.org/static/policies.action#sharing) requires authors to make all data underlying the findings described in their manuscript fully available without restriction, with rare exception (please refer to the Data Availability Statement in the manuscript PDF file). The data should be provided as part of the manuscript or its supporting information, or deposited to a public repository. For example, in addition to summary statistics, the data points behind means, medians and variance measures should be available. If there are restrictions on publicly sharing data—e.g. participant privacy or use of data from a third party—those must be specified.

Reviewer #1: Yes

Reviewer #2: Yes

4. Is the manuscript presented in an intelligible fashion and written in standard English?

PLOS ONE does not copyedit accepted manuscripts, so the language in submitted articles must be clear, correct, and unambiguous. Any typographical or grammatical errors should be corrected at revision, so please note any specific errors here.

Reviewer #1: Yes

Reviewer #2: Yes

5. Review Comments to the Author

Please use the space provided to explain your answers to the questions above. You may also include additional comments for the author, including concerns about dual publication, research ethics, or publication ethics. (Please upload your review as an attachment if it exceeds 20,000 characters)

**Comments to the Author**

**Reviewer #1:**

This is an important study done during difficult times, among healthcare personnel and the work done is appreciated.

The study brings out the main point that the exposure in this healthcare setting pointed toward community rather than occupational risk. Some comments are:

1. Since the study reports a prevalence value among healthcare staff, it may be important to mention that since the Nucleocapsid antigen was used, there could be underestimation of the prevalence as compared to the assays using the Spike protein as per some reports (eg: Changes in SARS-CoV-2 Spike versus Nucleoprotein Antibody Responses Impact the Estimates of Infections in Population-Based Seroprevalence Studies | Journal of Virology ([asm.org](http://asm.org/))).

**A: We thank the Reviewer for this observation. We have included an additional paragraph in the Discussion to mention this point as a limitation of the study: “Finally, as discussed in a recently published study [43], using anti-N antibody tests for population-based seroprevalence studies may result in the underestimation of prevalence due to waning of these antibodies over time in comparison with anti-S antibodies. This is a limitation of our study, although the impact of this observation in our setting would need to be assessed given that the first cases of SARS-CoV-2 infection in Mexico were reported in March 2020 and enrollment in our study began in October 2020, coinciding with the onset of the second wave that significantly increased infection incidence [1, 5]. Thus, we would expect a high proportion of the detected infections at baseline to be relatively recent. On the other hand, measuring anti-N antibodies allows to distinguish previously infected individuals from vaccinated individuals (for most approved vaccines). This is important given that vaccination efforts for healthcare personnel in Mexico started by the end of 2020. Nevertheless, even considering a possible underestimation, the prevalence rate was notably high in the examined population.”**

2. Although lab technicians are stated to be associated with higher risk at the first visit, and this is mentioned as a significant result in the abstract, no details are available about the specific type of work done related to exposure (sample collection or testing etc), use and availability of PPE etc for this group and on the possible reasons for the same especially when compared to doctors and nurses. Or was it a selection bias alone?

**A: The specific type of work of laboratory technicians was not collected as part of the metadata. However, we do have information on access to PPE, contact with COVID-19 patients and handling of biological specimens from COVID-19 patients (S3 Table). The self-reported frequency of biological specimen handling and contact with COVID-19 patients among laboratory technicians was not significantly different to that reported by nurses and physicians and thus, we would expect a higher significance of community-associated risk. However, this observation could also reflect selection bias in this group. Indeed, the frequency of previous COVI-19 diagnosis in laboratory technicians was higher than in physicians, nurses and administrative staff, suggesting a higher enrollment of persons interested in the results of the antibody tests for participants of this occupational group. This has been added to the Discussion.**

3. Line 181: In the models, participants
“182 were censored at the time of their last test, when they did not complete the originally planned 183 five tests and had negative results in all their samples.”
This statement is not clear. Were they removed completely?

**A: All the participants were included in the prevalence analysis. Participants with a positive result in their first sample were excluded from the incidence analysis and were considered prevalent cases. Participants who did not complete the five follow-up visits contributed person-time from all the available tests with negative results, until the first positive result or until their last available negative result. We have included the following sentence in the text to improve clarity: “In the models, participants with negative results in all their follow-up samples were censored at the time of their last available test.”**

4. Line 321 – The use of the word “posterior “is confusing. Does it mean “behind/before” or after?

**A: We have reworded the sentence in order to make it clearer: “This study comprises a later time period to that reported in other studies with similar and lower prevalence [8-10, 33].”**

**Reviewer #2:**

Abstract
• Details regarding the study participants can be added
• consent for participation and regular blood testing can be added
• Study duration and frequency of the blood tests done can be added
• What is the target sample size?

**A: Considering word limit, the abstract has been modified to include the details requested by the Reviewer. Study duration and frequency of blood tests were already included in the original version.**

Introduction
• Information on the reported burden (how many cases were reported during the first wave) and rationale of seroprevalence can be linked
• More content needs to be added on the need and process of conducting seroprevalence studies.
• Need a clear description on what is the knowledge/ research gap and how current study tries to address it.

**A: We believe that the original version of the manuscript already includes several of the points raised by the Reviewer to explain the rationale of the study as well as the knowledge gap being addressed:**

1. **Higher risk of exposure among health workers than the general population: Lines 77-79**
2. **Association of exposure with activities at work and working conditions: Lines 81-84**
3. **Wide variation in reported seroprevalence in healthcare workers across studies worldwide: Lines 84-86.**
4. **Unknown seroprevalence for SARS-CoV-2 in healthcare workers in the Mexican setting: Lines 102-103.**
5. **Advantages of conducting seroprevalence studies in healthcare workers to better understand occupational hazards and improve prevention measures: Lines 97-102**

**In order to improve this justification, we have added some additional content to the Introduction, as suggested, including an update of the epidemiological background (lines 67-71) and rationale of the study (lines 104-106; lines 110-112).**

Methods
• The rationale for conducting this study is not clearly mentioned anywhere, especially What is the rationale of including all types of health workers, did the authors assume that the risk was same for all the workers. If it is different risk, how did they analyze the sample size for each risk level?

**A: We believe that the original version of the manuscript includes several points to explain the rationale of the study, as detailed above.**

**In order to improve this justification, and following the Reviewer’s suggestion, we have added additional text in the Methods, explaining the rationale for including all types of healthcare workers: “We invited all healthcare workers of the participating institutions to enroll, regardless of occupation (staff physicians, physicians in training, nurses, laboratory technicians, researchers, students, housekeeping, maintenance, security and administrative staff). We included all occupations, given the hypothesized overall increased risk of exposure in the working environment due to the concentration of severely ill persons in the participating institutions, as well as the possibility of frequent contact of co-workers with different risk of exposure to SARS-CoV-2 according to their activities. Participants were enrolled between October 2020 and June 2021 and followed-up for up to five visits, separated at least 21 days one from each other. We collected blood samples for antibody testing and applied structured questionnaires for sociodemographic and occupational and community risk exposure information at each visit. We evaluated the risk of exposure to SARS-CoV-2 using multivariable models including the type of occupation and potential community and work risk variables.”**

**Sample size for different risk levels according to occupation were not calculated. Instead, we analyzed the independent and potential association of each work and community risk variable (including occupation) with SARS-CoV-2 exposure using multivariable logistic models. Stratifying by occupation was not possible, given the low number of participants in some categories.**

• Who had access to CASI data? How was the data security maintained?

**A: We have added the following information to the “Data collection” section of the Methods: “Data was stored in a local secured server. Only key research personnel and IT staff had access to the data and databases used for the analyses were previously de-identified. Each participant had access to a portal containing their individually scheduled appointments and the results from serological tests. Data privacy and access policies were explained to and accepted by the participants during the informed consent process and before answering the CASI”.**

• Line no. 146: please add reference number of approval from ethical committee

**A: An Ethics Statement section has been added, including registry numbers of the Institutional Review Boards, as well as reference numbers of the approved protocol.**

Results:
• Line number 233 to 235
• It is not clear as to whether it is a Figure title or started with explanation?

**A: We apologize for the confusion. The Journal requires Figure Legends to be inserted across the manuscript. This paragraph includes the title and legend for Figure 1. We have corrected formatting issues in order to make it more understandable.**

• What do you mean by prevalent cases as written in line number 250? The description of prevalent cases is given only in line number 275. It would be better to add it in line number 250 for better clarity

**A: We define prevalent, incident and non-cases in the Methods (Statistical analyses section). For better clarity, we have added the definition in the suggested line: “Considering only prevalent cases (persons with a positive antibody test result in their first visit; see Methods)”**

• Line No (255-262): Too lengthy sentence and not clear
• Table 2: What are the factors adjusted for Table 2? Please mention adjusted variables in the footnote of the table

**A: We have reworded the paragraph, separating sentences to improve clarity. We have added a footnote in Tables 2 and 3, explaining that only variables shown in the table were included in the multivariable models. This information is also included in the Statistical Analysis section of the Methods.**

Discussion
• Line No 343.: What are the reasons for lower participation rate in both the study centers?

**A: The reasons for low participation rate and its impact as a possible bias in the study are discussed in the original manuscript (lines 362-373 of the revised version). This section of the text was kept as originally written in the revised version: “Participation in the study was much lower than expected, with only 45% (548/1,211) and 14% (335/2,428) of the registered personnel of INER and INCMNSZ enrolled. This could be attributable to lack of interest in participating, little time due to high work burden, competing research protocols, problems accessing the electronic portal because of poor computational skills or lack of information on the current work (even after multiple communication efforts to promote the study). Indeed, we observed some differences between persons who did not attend (n=246) versus those who attended (n=883) their appointment for blood collection after registering in the study portal, including lower age, higher percentage of workers from INCMNSZ, higher proportion of nurses, higher proportion of persons that reported handling biological specimens, having frequent contact with patients with COVID-19, and using public transport (*P*<0.05 in all cases; S5 Table).“**

• How the data privacy of the health care staff was maintained across the study centers? This might be one of the reasons for non-participation too. How does the study overcome this issue? Please describe.

**A: We do not believe that data privacy could be a reason for non-participation. The databases compiling information about the participating healthcare personnel were only available for key research personnel and IT staff and databases for the analyses were previously de-identified. Data privacy and access policy were stated in the informed consent and before providing access to the CASI in the portal. All participants agreed with data privacy policies before providing answers. These points have been added to the “Data collection” section of the Methods.**

• Line number 233 to 235: It is not clear as to whether it is a Figure title or started with explanation?

**A: We apologize for the confusion. As explained above, the Journal requires Figure Legends to be inserted across the manuscript. This paragraph includes the title and legend for Figure 1. We have corrected formatting issues in order to make it more understandable.**

6. PLOS authors have the option to publish the peer review history of their article ([what does this mean?](https://journals.plos.org/plosone/s/editorial-and-peer-review-process#loc-peer-review-history)). If published, this will include your full peer review and any attached files.

If you choose “no”, your identity will remain anonymous but your review may still be made public.

**Do you want your identity to be public for this peer review?** For information about this choice, including consent withdrawal, please see our [Privacy Policy](https://www.plos.org/privacy-policy).

Reviewer #1: No

Reviewer #2: No
