# Supplementary material for: Seroepidemiology of SARS-CoV-2 in healthcare personnel working at the largest tertiary COVID-19 referral hospitals in Mexico City
Source: PLoS One. 2022 Mar 17;17(3):e0264964. doi: 10.1371/journal.pone.0264964 (PMC8929624; doi:10.1371/journal.pone.0264964)
Supplement: Attachment — Submitted filename: Response to Reviewers.docx [file pone.0264964.s009.docx]

Journal Requirements:

Please review your reference list to ensure that it is complete and correct. If you have cited papers that have been retracted, please include the rationale for doing so in the manuscript text, or remove these references and replace them with relevant current references. Any changes to the reference list should be mentioned in the rebuttal letter that accompanies your revised manuscript. If you need to cite a retracted article, indicate the article’s retracted status in the References list and also include a citation and full reference for the retraction notice.

[Note: HTML markup is below. Please do not edit.]

**A: References have been reviewed.**

Reviewers' comments:

Reviewer's Responses to Questions

**Comments to the Author**

1. If the authors have adequately addressed your comments raised in a previous round of review and you feel that this manuscript is now acceptable for publication, you may indicate that here to bypass the “Comments to the Author” section, enter your conflict of interest statement in the “Confidential to Editor” section, and submit your "Accept" recommendation.

Reviewer #1: (No Response)

Reviewer #2: All comments have been addressed

2. Is the manuscript technically sound, and do the data support the conclusions?

The manuscript must describe a technically sound piece of scientific research with data that supports the conclusions. Experiments must have been conducted rigorously, with appropriate controls, replication, and sample sizes. The conclusions must be drawn appropriately based on the data presented.

Reviewer #1: Yes

Reviewer #2: Yes

3. Has the statistical analysis been performed appropriately and rigorously?

Reviewer #1: Yes

Reviewer #2: Yes

4. Have the authors made all data underlying the findings in their manuscript fully available?

The [PLOS Data policy](http://www.plosone.org/static/policies.action#sharing) requires authors to make all data underlying the findings described in their manuscript fully available without restriction, with rare exception (please refer to the Data Availability Statement in the manuscript PDF file). The data should be provided as part of the manuscript or its supporting information, or deposited to a public repository. For example, in addition to summary statistics, the data points behind means, medians and variance measures should be available. If there are restrictions on publicly sharing data—e.g. participant privacy or use of data from a third party—those must be specified.

Reviewer #1: Yes

Reviewer #2: Yes

5. Is the manuscript presented in an intelligible fashion and written in standard English?

PLOS ONE does not copyedit accepted manuscripts, so the language in submitted articles must be clear, correct, and unambiguous. Any typographical or grammatical errors should be corrected at revision, so please note any specific errors here.

Reviewer #1: Yes

Reviewer #2: Yes

6. Review Comments to the Author

Please use the space provided to explain your answers to the questions above. You may also include additional comments for the author, including concerns about dual publication, research ethics, or publication ethics. (Please upload your review as an attachment if it exceeds 20,000 characters)

Reviewer #1: All comments except 3 are fully addressed.
Regarding the earlier comment 3.
Line 181: In the models, participants “182 were censored at the time of their last test, when they did not complete the originally planned 183 five tests and had negative results in all their samples.” This statement is not clear. Were they removed completely?

A: All the participants were included in the prevalence analysis. Participants with a positive result in their first sample were excluded from the incidence analysis and were considered prevalent cases. Participants who did not complete the five follow-up visits contributed person-time from all the available tests with negative results, until the first positive result or until their last available negative result. We have included the following sentence in the text to improve clarity: “In the models, participants with negative results in all their follow-up samples were censored at the time of their last available test.”

From the response to the earlier comment above, it appears that the person time included the time till their last available negative visit if they did not complete five visits.
"Participants who did not complete the five follow-up visits contributed person-time from all the available tests with negative results, until the first positive result or until their last available negative result."

“In the models, participants with negative results in all their follow-up samples were censored at the time of their last available test.”
But does “censored” mean that they were removed? It is confusing if they were removed, whether only the last test was removed and also why they were removed. Or were they removed for calculation of the incident cases?

**A: In this version, we eliminated the term “censored”, which is different to “removed” to avoid any confusion. Instead, we simply describe how we made the calculations. We planned five follow-up visits for blood sample donation for every participant, but only a fraction of the participants complied with the five visits. The test result of the first visit for every participant was used to define prevalent cases. Participants that resulted positive were included in the seroprevalence calculation.**

**Prevalent cases were excluded from incidence estimations. All participants included in the incidence calculation contributed with follow-up time from the date of the first sample until the date of the first sample with a positive result (incident cases), or until the last date of follow up (non-cases).** **Please see the definition of the prevalent, incident and non-case groups, in lines 185-188. We have included the following text in the Statistical analyses section: “The person-time was calculated as follows: incident cases contributed with the time elapsed from the date of their first sample to the date of their first positive sample, and non-cases contributed with their complete follow-up time from their first to their last sample date.”**

Additional comment 1:
At the end of the study, 290 participants had a positive result in any of the antibody tests, yielding 241 an overall prevalence in the study period, adjusted by sensitivity and specificity of the antibody
Line 242 test, of 33.5%. 235 positive cases (81.9%) were identified at baseline (prevalent cases), while the 243 remaining 55 (6.2%) seroconverted during the follow up period (incident cases).
Among the positive cases (290) – the prevalent cases were 235/290 = 81.9%, while the incident cases would be 55/290 which is 18.9 % and not 6.2% as mentioned – is that correct?

**A: Thank you for pointing out this error. We have changed the text to 18.9%, to show the percentage relative to the total positive cases.**

Additional comment 2: Line 68 - spelling error "waive" caused by the omicron variant.

**A: The mistake has been corrected.**

Reviewer #2: Thanks for addressing the earlier comments.
I encourage the authors to please check the manuscript for grammar and sentence structuring before final proof is sent.

**A: We thank the Reviewer for the comments. The manuscript has been checked for grammar and structure with the appropriate corrections.**

7. PLOS authors have the option to publish the peer review history of their article ([what does this mean?](https://journals.plos.org/plosone/s/editorial-and-peer-review-process#loc-peer-review-history)). If published, this will include your full peer review and any attached files.

If you choose “no”, your identity will remain anonymous but your review may still be made public.

**Do you want your identity to be public for this peer review?** For information about this choice, including consent withdrawal, please see our [Privacy Policy](https://www.plos.org/privacy-policy).

Reviewer #1: No

Reviewer #2: **Yes:** Giridhara R Babu
